# A human, compact, fully functional anti-ErbB2 antibody as a novel antitumour agent

**DOI:** 10.1038/sj.bjc.6602110

**Published:** 2004-08-10

**Authors:** C De Lorenzo, A Tedesco, G Terrazzano, R Cozzolino, P Laccetti, R Piccoli, G D'Alessio

**Affiliations:** 1 Department of Biological Chemistry, University of Naples Federico II, Via Mezzocannone 16, 80134 Naples, Italy; 2 Department of Cellular and Molecular Biology and Pathology ‘L Califano’, University of Naples Federico II, via Pansini 5, 80131 Naples, Italy

**Keywords:** immunotherapy, antibody engineering, ErbB2, mammary carcinoma

## Abstract

A new human, compact antibody was engineered by fusion of a human, antitumour ErbB2-directed scFv with a human IgG1 Fc domain. Overexpression of the ErbB2 receptor is related to tumour aggressiveness and poor prognosis. This new immunoagent meets all criteria for a potential anticancer drug: it is human, hence poorly or not immunogenic; it binds selectively and with high affinity to target cells, on which it exerts an effective and selective antiproliferative action, including both antibody-dependent and complement-dependent cytotoxicity; it effectively inhibits tumour growth *in vivo*. Its compact molecular size should provide for an efficient tissue penetration, yet suitable to a prolonged serum half-life.

Immunotherapy represents an effective strategy to fight cancer, mainly based on antibodies specifically directed to selected cancer cells (Trikha *et al*, 2002; Ross *et al*, 2003). Obstacles to full success of present-day immunotherapy include: (i) immune responses against non-human, or even humanised antibodies (Kuus-Reichel *et al*, 1994); (ii) the large size of antibodies, which hinders their diffusion into bulky tumours. Fully human, small-sized antitumour immunoagents would overcome these risks, and provide safe, highly selective and effective antitumour drugs.

A good candidate as a tumour-associated antigen, and an attractive target for immunotherapy, is ErbB2 (also known as Her-2/Neu), a transmembrane tyrosine kinase receptor, overexpressed in breast carcinomas, for which it is a marker of poor prognosis (Slamon *et al*, 1987). We recently isolated a novel human anti-ErbB2 single-chain variable fragment (scFv) from a large phage-display library (Griffin 1) through a double selection strategy performed on live cells (De Lorenzo *et al*, 2002). This scFv, named Erbicin, specifically binds to ErbB2-positive cells, inhibits the receptor autophosphorylation, is internalised in target cells and strongly inhibits their proliferation (De Lorenzo *et al*, 2002). However, the small size of the scFv and its expected rapid clearance from the bloodstream, as well as its monovalent nature, could limit its use as a therapeutic agent selective for mammary carcinoma cells.

A significant addition to the anticancer arsenal would be the construction of a new anti-ErbB2 immunoagent from a human, *per se* cytotoxic scFv and a human Fc domain. This fully human antitumour antibody would be a compact, reduced version of an IgG, with the antiproliferative effect of the scFv moiety on tumour target cells, combined with the ability of the Fc moiety to induce both antibody-dependent cellular cytotoxicity (ADCC) and complement-dependent cytotoxicity (CDC). Yet, such a compact antibody is expected to have (Demignot *et al*, 1990; Yokota *et al*, 1993; Powers *et al*, 2001) a more protracted half-life and higher tumour retention than the parental scFv, but improved penetration properties in solid tumours with respect to full-size IgG agents.

## MATERIALS AND METHODS

### Cell cultures and antibodies

The SKBR3 cell line from human breast cancer, the A431 cell line from human epidermoid carcinoma, and Chinese hamster ovary (CHO) cells (all from American Type Culture Collection, Rockville, MD, USA) were cultured in RPMI 1640 (Gibco BRL, Life Technologies, Paisley, UK). The TUBO cell line from a BALB-neu T mouse-derived mammary lobular carcinoma (kindly provided by Dr G Forni, University of Turin, Italy) was grown in DMEM (Gibco BRL). The media were supplemented with 10% foetal bovine serum (20% for TUBO cells), 50 U ml^−1^ penicillin, and 50 *μ*g ml^−1^ streptomycin (all from Gibco BRL).

The antibodies used were: Herceptin (Genentech, South San Francisco, CA, USA); monoclonal anti-human IgG1 (Fc specific, Sigma, St Louis, MO, USA); horseradish peroxidase-conjugated goat anti-mouse immunoglobulins (Pierce, Rockford, IL, USA); horseradish peroxidase-conjugated goat anti-human affinity-isolated IgG (Fc specific) (Sigma).

### Peripheral blood lymphocytes

Peripheral blood lymphocytes (PBL) were obtained from peripheral blood mononuclear cells (PBMC) isolated by centrifugation on Lymphoprep gradients (Axis Shield PoC AS, Oslo, Norway) from normal donor buffy coats obtained from the Blood Bank of the Medical School of the University of Naples ‘Federico II’. After the separation, PBL were washed twice and incubated in RPMI 1640 medium (Gibco BRL) for 2 h at 37°C to remove adherent cells. The nonadherent cells were used as natural cytotoxic effectors without any additional treatment.

### Construction and production of the anti-ErbB2 Erb-hcAb

In a previous paper (De Lorenzo *et al*, 2002), we reported the isolation of a novel human anti-ErbB2 scFv, selected by panning the Griffin 1 phage library on live cells with a subtractive selection strategy based on two combinations of ErbB2-positive and -negative cell lines. The cDNA coding for the anti-ErbB2 scFv was amplified from vector pHEN2 by PCR using as forward and reverse primers oligonucleotides containing at their 5′ end a *Hin*dIII site and a *Bam*HI site, respectively: (5′-CCCCCCAAGCTTCAGGTGCAGCTGTTG-3′; 5′-AACCGCGGATCCGCACCTAGGACGGTCAG-3′). The PCR fragment was then digested with *Hin*dIII and *Bam*HI (New England Biolabs, Hertfordshire, UK) for cloning into the corresponding sites in plasmid pIg1plus (R & D Systems, Minneapolis, USA), downstream to the leader sequence and upstream to the hinge-CH2-CH3 sequence, respectively, of a human IgG1 heavy chain constant region (Fc).

The fusion protein was produced by transfecting CHO cells with the recombinant vector. In brief, cells grown in RPMI containing 10% FCS at 70–80% confluency were transfected with 5 *μ*g of expression vector using the Superfect reagent (Qiagen, Valencia, CA, USA). Stable transfectants were selected in the presence of G418 (Sigma) at a concentration of 1 mg ml^−1^. Expression of the antibody construct was determined in the culture medium by quantitative ELISA. For recombinant protein production, transfected CHO cells were expanded to near confluence in selective medium containing neomycin, and then were grown for 3–4 days in serum-free medium.

The recombinant fusion protein, henceforth termed Erb-hcAb, secreted by transfected CHO cells, was purified from culture medium by affinity chromatography on a protein A-Ceramic Hyper D®F column (BioSepra, Cergy-Saint-Christophe, France) loaded with 300–500 ml of conditioned medium, washed with 10 volumes of 100 mM Tris-HCl, pH 8.0 containing 0.5 M NaCl, and 10 volumes of 10 mM Tris-HCl, pH 8.0. The protein eluate was obtained with 50 mM glycine pH 3.0, and immediately neutralised with 1/10 volume of 1 M Tris-HCl, pH 8.0.

### ELISA assays

ErbB2-positive SKBR3 cells and ErbB2-negative A431 control cells, harvested in nonenzymatic dissociation solution (Sigma), were washed and transferred to U-bottom microtitre plates (1 × 10^5^ cells per well). After blocking with PBS containing 6% bovine serum albumin (BSA), cells were incubated with conditioned medium or purified immunoagents in ELISA buffer (PBS/BSA 3%) for 90 min. The pelleted cells were washed, resuspended in 100 *μ*l of ELISA buffer, and incubated with an anti-human IgG (Fc-specific) mAb (Sigma) or anti-myc mAb 9E10 (Evan *et al*, 1985), for detection of Erb-hcAb and Erbicin (containing the myc tag), respectively. Goat anti-mouse HRP-conjugated immunoglobulins (Pierce, Rockford, IL, USA) were used for detection of bound antibodies. After 1 h, the plates were centrifuged, washed with ELISA buffer, and reacted with 3,3′,5,5′-tetramethylbenzidine (TMB) (Sigma). Binding values were determined from the absorbance at 450 nm, and reported as the mean of at least three determinations (s.d. ⩽5%).

### Cell growth inhibition assays

Cells were seeded in 96-well, flat-bottom plates; SKBR3 and TUBO cells at a density of 1.5 × 10^4^ well; A431 at a density of 5 × 10^3^ well. After addition of the protein under test viable cells by the Trypan blue-exclusion test were counted at suitable time intervals. Cell survival was expressed as percent of viable cells in the presence of the protein under test with respect to control cultures grown in the absence of the protein. Typically, cell survival values were obtained from at least three separate experiments in which triplicate counts were determined; standard deviations were below 5%.

### ADCC and CDC tests

Target and control cells were detached from culture dishes with a cell dissociation solution (Sigma) and transferred to round-bottom 96-well plates (2 × 10^4^ cells per well). For ADCC assays, target or control cells were treated with the immunoagents (3 *μ*g ml^−1^ of serum-free medium) and peripheral blood lymphocytes (PBL) at 37°C for 3–4 h. For CDC assays, cells were incubated at 37°C with human serum. Cultures were performed in triplicate in a final volume of 200 *μ*l. Controls included target cells incubated in the absence of effector, or in the presence of either serum or immunoagent alone. Tumour cell lysis was determined by measuring the release of lactate dehydrogenase (LDH) using a LDH detection kit (Roche, Mannheim, Germany). ADCC or CDC were calculated as the percent of cytolysis measured in the presence of immunoagent and PBL or human serum, for ADCC and CDC, respectively, taking as 100% the maximal LDH release determined by lysis of target cells with 1% Triton X-100.

### *In vivo* antitumour activity

All experiments were performed with 6-week-old female Balb/cAnNCrlBR mice (Charles River Laboratories, Calco, Italy). TUBO cells (5 × 10^5^) were suspended in 0.2 ml sterile PBS and injected subcutaneously (day 0) in the right paw. At day 7, when tumour started to appear, the mice were divided into two groups. At day 15, when tumours were clearly detectable, Erb-hcAb dissolved in PBS was administered at a site remote from that of tumour implantation, at doses of 2.5 mg kg^−1^ of body weight for 7 times at 72 h intervals. The second group of control animals was treated with identical volumes of sterile PBS. During the period of treatment, tumour volumes (*V*) were measured with caliper and calculated by the formula of rotational ellipsoid *V*=*A* × *B*^2^/2 (*A* is the axial diameter, *B* the rotational diameter). All mice were maintained at the animal facility of the Department of Cellular and Molecular Biology and Pathology, University of Naples Federico II. Animal studies were conducted in accordance with the Italian regulation for experimentation on animals. All *in vivo* experiments were carried out with ethical committee approval and met the standards required by the UKCCCR guidelines (Workman *et al*, 1998).

## RESULTS

### Construction and characterisation of a human anti-ErbB2 compact antibody

**Figure 1:**
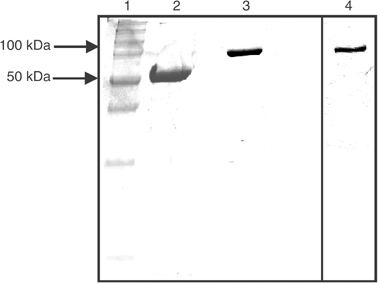
SDS–PAGE of purified Erb-hcAb. Erb-hcAb was run under reducing (lane 2) and nonreducing (lane 3) conditions. Lane 4: Western blot analysis of the sample run in lane 3 tested with an anti-human IgG1 (Fc specific) antibody. Molecular weight standards are in lane 1.

We generated a new anti-ErbB2 immunoagent by fusing the scFv Erbicin with a human IgG1 Fc domain. The IgG-like protein has a reduced molecular weight, and contains all functionally relevant antibody regions. The cDNA coding for the human anti-ErbB2 scFv was amplified by PCR and cloned into a eukaryotic expression vector containing the human IgG1 Fc sequence. The recombinant plasmid, sequenced to confirm faithful cloning, was stably transfected in Chinese hamster ovary cells, and expressed as a secretion product into the culture medium. After purification by affinity chromatography, the final yield of the fusion protein was 1.5 mg l^−1^. The immunoagent was named Erb-hcAb (anti-ErbB2 human compact antibody). When Erb-hcAb was analysed by SDS–PAGE (Figure 1

), it was found to migrate under reducing conditions with the expected molecular size of about 50 kDa, and as a dimer of about 100 kDa under nonreducing conditions. This indicated that the fusion protein is expressed as a disulphide-linked dimer. Western blotting analysis performed with an anti-human Fc mAb demonstrated immunoreactivity of the purified, dimeric protein with 100 kDa size (Figure 1).

**Figure 2:**
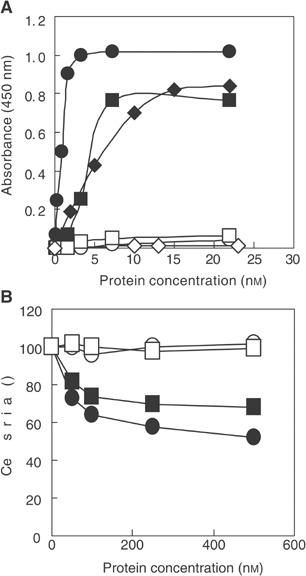
Effects of Erb-hcAb on ErbB2-positive (SKBR3) and ErbB2-negative (A431) cells. (**A**) ELISA assays of SKBR3 cells (black symbols) and A431 cells (empty symbols) tested with Erb-hcAb (circles), the parental anti-ErbB2 scFv (rhomboids), or Herceptin (squares). (**B**) Dose–response curves for ErbB2-positive SKBR3 cells (black symbols), or ErbB2-negative A431 cells (empty symbols), treated for 72 h with Erb-hcAb (circles), or Herceptin (squares).

When the ability of the recombinant fusion protein to bind ErbB2-positive cells was analysed by ELISA assays (Figure 2A

), Erb-hcAb was found to fully retain the specificity of the original scFv for mammary carcinoma SKBR3 ErbB2-overexpressing cells. It did not react instead with ErbB2-negative cells such as A431 cells (from human epidermoid carcinoma), which overexpress the ErbB1 EGF receptor from the same ErbB family. The apparent binding affinity of the compact antibody for the ErbB2 receptor, that is, the concentration corresponding to half-maximal saturation, was about 1 nM, comparable to the values of 4 and 5 nM, determined for the parental scFv, and Herceptin, a humanised anti-ErbB2 monoclonal, respectively (see Figure 2A). Presumably, the four-fold increased avidity with respect to Erbicin is due to the acquired bivalency of Erb-hcAb for ErbB2.

### Biological effects of Erb-hcAb on ErbB2-positive tumour cells

The effect of Erb-hcAb on tumour cell growth was assessed by measuring the survival of SKBR3 cells treated with increasing concentrations of Erb-hcAb. As shown in Figure 2B, the anti-ErbB2 Erb-hcAb inhibited the growth of SKBR3 cells in a dose-dependent fashion, with an antiproliferative effect more pronounced than that observed for Herceptin, and no effects on the proliferation of ErbB2-negative A431 cells (see Figure 2B).

**Figure 3:**
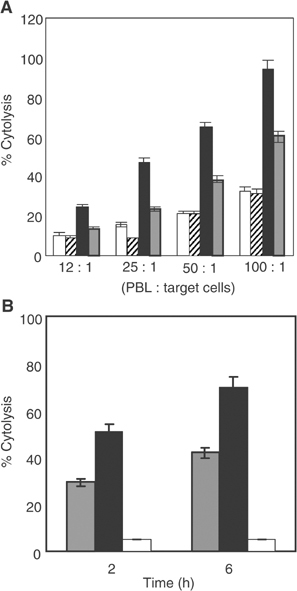
Antibody-dependent and complement-dependent cytotoxicity assays of Erb-hcAb. (**A**) SKBR3 cells treated with PBL as effector cells at four different ratios: in the absence (white bars), or in the presence of Erb-hcAb (black bars). Erbicin, the parental anti-ErbB2 scFv (striped bars), and Herceptin (grey bars) were used as a negative and a positive control, respectively. (**B**) ErbB2-overexpressing SKBR3 cells were incubated for 2 or 6 h, in the presence of human serum as a source of complement, with Erb-hcAb at a concentration of 3 *μ*g ml^−1^ (shaded bars) or 10 *μ*g ml^−1^ (black bars). Herceptin (10 *μ*g ml^−1^) was used as a negative control (white bars).

To investigate whether Erb-hcAb was capable of recruiting immune effector functions *in vitro*, assays for cytolysis of tumour cells as induced by PBL, or complement, were performed. To determine the capacity of Erb-hcAb to trigger ADCC towards antigen-expressing cells, ErbB2-positive and ErbB2-negative control cells were incubated for 3 h with increasing amounts of effector PBL in the absence or in the presence of Erb-hcAb (3 *μ*g ml^−1^). As shown in Figure 3A

, Erb-hcAb effectively lysed SKBR3 target cells in the presence of PBL. The extent of lysis reached almost 100% of treated cells, whereas Herceptin, used as a positive control, induced about 60% lysis. The basal level of cytotoxicity was measured in the presence of PBL (see Figure 3A) or Erb-hcAb alone (data not shown). No effects were detected in parallel assays carried out with ErbB2-negative cells, such as A431 (data not shown), or when Erb-hcAb was replaced by the parental anti-ErbB2 scFv, lacking the Fc domain (see Figure 3A). These results indicate the specificity of the Erb-hcAb-dependent cell-mediated cytolytic activity, clearly based on both binding abilities of the immunoagent: (i) to the cognate receptor with its antigen binding sites; (ii) to natural killer cells with its Fc effector domain.

To test the ability of Erb-hcAb to induce CDC against ErbB2-positive tumour cells, SKBR3 target cells were incubated for 2 or 6 h with Erb-hcAb (at 3 or 10 *μ*g ml^−1^ concentrations) in the absence or the presence of human serum as a source of complement. As illustrated in Figure 3B, Erb-hcAb was found to effectively lyse SKBR3 cells in the presence of serum, with an average specific lysis of 50% after 2 h, increased to about 70% after additional 4 h of incubation with 10 *μ*g ml^−1^ of Erb-hcAb (see Figure 3B). Heat inactivation of serum completely abolished the complement-dependent lysis. Moreover, CDC was not detected when ErbB2-negative A431 cells were incubated with Erb-hcAb and human serum (data not shown). Similarly, as expected (Drebrin *et al*, 1988), no lysis was detectable when SKBR3 cells were treated with Herceptin in the presence of human serum (see Figure 3B).

### *In vivo* antitumour activity of Erb-hcAb

For *in vivo* studies, Erb-hcAb was tested on murine TUBO tumour cells expressing ErbB2 of rat origin (Rovero *et al*, 2000). *In vitro*, TUBO cells were found to be sensitive to the treatment with Erb-hcAb with an IC_50_ of 0.5 *μ*M. This indicated cross-reactivity between the human immunoagent and rat ErbB2 (Rovero *et al*, 2000). This may not be surprising, as the structures of the two homologs are highly superimposable (Cho *et al*, 2003).

**Figure 4:**
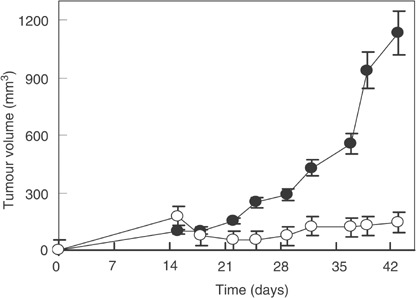
*In vivo* suppression of tumour growth by Erb-hcAb. Tumour growth was followed in mice inoculated s.c. with 5 × 10^5^ TUBO mammary carcinoma cells. Control animals (black circles) were treated with sterile PBS solution. Treated animals (white circles) were injected with Erb-hcAb, starting at day 15. Seven doses, each of 2.5 mg kg^−1^ of body weight, were administered at 72 h intervals.

When administered to mice, TUBO cells induce tumours very similar to the alveolar-type human lobular mammary carcinomas (Di Carlo *et al*, 1999). As shown in Figure 4

, the treatment of mice bearing TUBO tumours with seven doses, at 72 h intervals, of 2.5 mg kg^−1^ of Erb-hcAb induced a dramatic reduction (96%) in tumour volume. During the period of treatment, the animals did not show signs of wasting or other visible signs of toxicity.

## DISCUSSION

The results described in this report show that the Erb-hcAb immunoagent has a high therapeutic potential, as it fully satisfies the conditions required for a successful anticancer agent: it is a fully human immunoagent, hence presumably with a reduced or no immunogenicity; it recognises with high affinity one of the most specific tumour-associated antigens, such as ErbB2; it displays effective antibody effector functions; it is effective in inhibiting target cell growth both *in vitro* and *in vivo*. Furthermore, its size should be better suited to therapeutic applications than either a small scFv, or full-size IgG-like molecules.

Previous reports have shown the feasibility of cloning single-gene constructs encoding fusion proteins made up of murine scFv and Fc fragments (Shu *et al*, 1993; Li *et al*, 2000; Xu *et al*, 2000). Recently, a recombinant, human scFv-Fc antibody has been reported (Powers *et al*, 2001) to mediate *in vitro* ADCC and endure a much longer serum half-life *in vivo* when compared to its parental scFv. However, the protein was produced in yeast with yeast-controlled glycosylation; furthermore, it was found to be heterogeneous, obtained in very low yields, and only partially glycosylated. It should be noted that Erb-hcAb, as reported above, was prepared instead in CHO cells, a mammalian model certainly closer than yeast to human cells.

Herceptin, currently used for treatment of advanced breast cancer (Baselga *et al*, 1999; Stebbing *et al*, 2000), is a humanised version of a murine anti-ErbB2 antibody. Its antitumour activity is mostly based on its ability to downregulate ErbB2 and induce ADCC (Sliwkowski *et al*, 1999), but, as previously reported (Drebrin *et al*, 1988), it does not elicit CDC. The new immunoagent Erb-hcAb instead displays a strong CDC effect, and has a reduced molecular size (100 kDa) with respect to that of Herceptin (155 kDa). It has been shown (Demignot *et al*, 1990; Yokota *et al*, 1993; Powers *et al*, 2001) that in an immunoagent of about 100 kDa the advantage of the prolonged half-life of an intact antibody is composed with an increased extravascular diffusion, both very expedient features for targeting solid tumours.

Taken together, the data reported here suggest that Erb-hcAb is a promising new anticancer agent, and supports the concept that, after humanised monoclonals and scFvs, a third generation of immunoagents, human compact antibodies, may represent the format of choice for the therapy of solid tumours.
